# Contact and SARS-CoV-2 Infections Among College Football Athletes in the Southeastern Conference During the COVID-19 Pandemic

**DOI:** 10.1001/jamanetworkopen.2021.35566

**Published:** 2021-10-29

**Authors:** Benika C. Dixon, Rebecca S. B. Fischer, Hongwei Zhao, Catherine S. O’Neal, James R. Clugston, Shawn G. Gibbs

**Affiliations:** 1 Department of Epidemiology & Biostatistics, School of Public Health, Texas A&M University, College Station; 2 Department of Medicine, Louisiana State University Health Sciences Center, Baton Rouge; 3 Department of Community Health & Family Medicine and Neurology, College of Medicine, University of Florida, Gainesville; 4 Department of Environmental and Occupational Health, School of Public Health, Texas A&M University, College Station

## Abstract

This cohort study of college football players in a single athletic conference examines the association of close contact events among players on opposing teams and subsequent positive SARS-CoV-2 tests.

## Introduction

The COVID-19 pandemic created heightened concern over SARS-CoV-2 transmission during athletic competitions and necessitated innovative mitigation solutions for protecting athletes, staff, and attendees.^1,2^ During sporting events, efforts like contact tracing pose unique challenges; namely, contact between athletes during play may be brief but recurring, while also challenging to track and triage, especially with interstate competitions.

When the National Collegiate Athletic Association declared football a high-risk transmission sport, the Southeastern Conference (SEC), an intercollegiate athletic conference of 14 universities in 11 southern US states, responded with protocols aligned with US Centers for Disease Control and Prevention (CDC) guidance to monitor, manage, and mitigate SARS-CoV-2 exposure.^3,4^ In part, traditional contact tracing was augmented using wearable, remote proximity loggers to document interpersonal contacts. In this cohort study, we analyzed SARS-CoV-2 contact exposures and transmission among opposing team players during college football games as the COVID-19 pandemic surged.

## Methods

The SEC implemented a system for remote sensing and automated logging of interactions and proximity between players wearing tracking devices (KINEXON).^5^ Athletes in the SEC underwent SARS-CoV-2 surveillance by polymerase chain reaction (PCR) testing at least 3 times per week, and athletes testing positive within 48 hours of game play were traced for potential exposures and subsequent infections over 14 days using the CDC close contact definition of 15 or more cumulative minutes spent within 6 feet of an infected person.

In this cohort study, we analyzed opposing player contact events (ie, within 6-feet proximity) recorded during official game times and SEC testing data for athletes in play. Analyses were done in Stata version 15 (StataCorp). The institutional review board at Texas A&M University reviewed this analysis and waived informed consent requirements because data were entirely deidentified.

## Results

**Figure 1.**
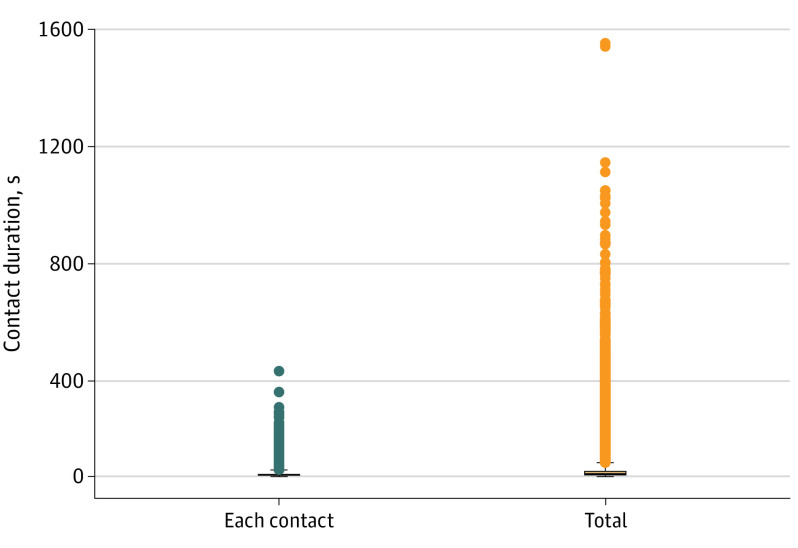
Duration of Close Proximity Contacts Between Players for Each Unique Opposing Player Pair During an SEC Game

Between September 26 and December 19, 2020, 1190 college football athletes had 109 762 opposing-player interactions over 64 SEC regular season games. Interactions were fleeting (median length, 6 seconds [range, 1-380 seconds]), and most (104 274 [95%]) were briefer than 26 seconds. Accumulated contact time between 2 players during a single game was fewer than 23 seconds (median length, 10 seconds [range, 1-1558 seconds]; 37 389 [95%] opponent pairs below 97 seconds) in most (29 518 [75%]) instances (Figure 1). Only 13 opponent-pairs had cumulative in-game contact longer than 15 minutes; none of the 13 tested positive for SARS-CoV-2 during 14 days pregame or postgame.

**Figure 2.**
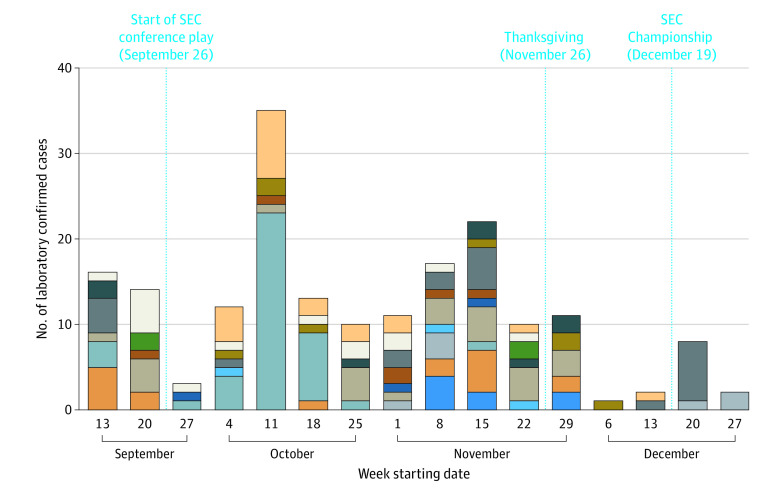
Laboratory Confirmed SARS-CoV-2 Infections by Southeastern Conference (SEC) Team During 2020 Season Each color represents a different SEC team—key intentionally excluded.

In all, 138 (11.6%) of the 1190 players tested SARS-CoV-2 positive (Figure 2), 18 (1.5%) of whom tested positive within 48 hours of playing in a game. Contact tracing revealed the 18 players had interactions with opponents lasting 1 to 364 seconds (median, 12 seconds), with no contacted athletes testing positive over the following 14 days; thus, none were considered close contact exposures under the current CDC definition.

## Discussion

In this analysis of US college athletes playing regular season SEC football games during the COVID-19 pandemic, no instances of in-game SARS-CoV-2 transmission was found. This is similar to the National Football League (NFL) and Super League reports, where game-related close contact exposure was not described.^5,6^ The SEC early-season peak SARS-CoV-2 incidence also mirrors the NFL experience. The rigorous SEC testing regime makes it unlikely that infections, even if asymptomatic or early in the infection period, went undetected.

We report low SARS-CoV-2 interteam transmission risk in college football when rigorous and multipronged protection strategies were implemented, suggesting game play during a pandemic did not seed outbreaks across jurisdictions. Despite 12% of athletes testing positive for SARS-CoV-2, only 18 had competed during the preceding 48 hours, and no downstream infections to members of the opposing team were apparent. Actual contacts were exceedingly brief, even when accumulated over an entire game. In-game close contact was rare, not associated with SARS-CoV-2 transmission, and no CDC defined close contact exposures were observed.

This study had several limitations. Further analysis of specific contact types or player positions, stopped clock or pregame and postgame periods, environmental conditions, and contact between teammates, referees, coaches, and other individuals onfield during games, along with clinical and public health case data, could shed additional light on SARS-CoV-2 infection and transmission among athletes. Implications of sporting events and other game-related activities, such as training and travel, on SARS-CoV-2 transmission in athletic organizations and communities warrant more extensive analysis.

Even during a pandemic, infectious diseases can be effectively monitored and prevented during contact sports through multipronged and innovative strategies that leverage traditional public health practices and applied technologies. Active and vigilant surveillance can prevent introduction of SARS-CoV-2 or similar threats into game play and prevent game-specific exposures, transmission, and downstream infections and reduce stress on public health systems.
